# The Mayor’s Gift to Buffalo

**Published:** 1910-01

**Authors:** 


### The Mayor's Gift to Buffalo

MR. J. N. Adam, who has served the city of Buffalo for the past four years as mayor, accentuated the ending of his official career by leaving a substantial legacy to the city. He presented a farm of 293 acres for a site for a tuberculosis hospital, valued at $19 560, located near Perrysburg in the County of Cattaraugus. It appears that sometime ago a commission was appointed to report on the proper location for such a hospital.

The Mayor made the announcement of his purpose during the executive session of the hospital commission in his office, December 10, 1909, immediately following the selection of the site in question as the most desirable of the 60 or 70 inspected by the commission.

When the vote was taken on the matter of choosing a site and was seen to be unanimous for the Perrysburg location, Mayor Adam said: "I will pay for it." The announcement came so suddenly that the other members of the committee wondered if they had heard aright. Then Mayor Adam added that he proposed, now that the choice had been made, to buy the site and give it to the city.

Mayor Adam on the sixteenth announced the appointment of a board of trustees to have charge of the building and management of the J. N. Adam Memorial Hospital, a retreat for sufferers from incipient tuberculosis, to be built on a site selected near Perrysburg, as already related.

The trustees named and their respective terms are as follows: John H. Pryor, six years; Thomas B. Lockwood, five years; William A. Douglas, four years; Roswell Park, three years; Maria M. Love, two years; Daniel J. Kenefick, one year. The ex-officio members of the board are the Mayor, President of the Common Council, and the Commissioner of Public Works.

The importance of this gift cannot be overestimated. It places Buffalo in the front rank of municipalities in the contest against tuberculosis. And it leaves the Mayor a distinct claim to the respect and affection of our citizens.

---

Dr. Thomas Jonnesco, of Bucharest, came to this country on the last day of November to demonstrate his method of spinal anesthesia for general surgical purposes. At a demonstration December 8, 1909, at the New York Hospital for Ruptured and Crippled several prominent surgeons were present. Dr. Virgil P. Gibney, Dr. William B. Coley, and Dr. Homer Gibney, were the operators. Next day four operations were made at the Post-Graduate Hospital, one by Dr. Jonnesco himself and the others by Dr. Robert T. Morris. The injection of stovaine combined with strychnine in these cases seemed to serve the purpose as well as a general anesthetic. Further tests however, would appear necessary before final judgment can be given.

---

A reception was given by the Manhattan Medical Society Friday evening, December 17, 1909, at 8.30, to Dr. John B. Deaver, of Philadelphia, at Reisenweber's, Eighth avenue and Fifty-eighth street New York. Dr. Deaver read a paper on "The Use and Abuse of Gastro-jejunostomy," which was discussed by Drs. Willy Meyer, Robert T. Morris, Carl Beck, J. F. Erdmann, of New York and G. K. Dickinson of Jersey City.

Dr. Deaver is one of the leading authorities on appendicitis in the United States. Not long ago a dinner was given to him in Philadelphia by about 150 physicians, upon many of whom he had operated for this condition.

---

ANNOUNCEMENT was made December 20, 1909, by the trustees of the University of Pennsylvania that Henry Phipps, founder of the Phipps Institute in Philadelphia, had presented to the university $500,000 to be used in the campaign against tuberculosis. The management of the Phipps institute will fall upon the university trustees, and the study, treatment, prevention of the disease will be continued in a new hospital to be erected.

---

GEORGE CROCKER'S gift to Columbia University to be used for the investigation of cancer will amount to at least $1,500,000, according to the terms of his will, made public recently. This sum will be realised by the sale of the city home and country estate.

---

THE Yale Alumni Weekly recently made formal announcement that an anonymous alumnus of Yale has offered a prize of $100,000 for the person who shall first discover an adequate remedy for tuberculosis. The prize fund has been placed in the custody of Yale University and the Yale medical school faculty are to act as its trustees. A number of the leading physicians of the country have been invited to become members of an advisory board, whose duty it shall be to pass on the merits of cures submitted.

The income from the fund is to be used for the investigation of any remedies which come to the attention of the trustees or members of the advisory board that have not been submitted for the prize. A condition of the award is that the cure under consideration shall have been in use for at least five years and during that time have proved its actual and unquestioned efficiency as a cure for tuberculosis.

---

THE Health Department of Buffalo has vacated the offices occupied since the erection of the Municipal Building on Delaware Avenue and moved to the quarters prepared for it at the corner of Church and Franklin streets, opposite the City Hall.

---

IT is not unusual for physicians to abbreviate the names of drugs used in prescriptions, partly to save space and time but largely to hide their ignorance of Latin grammar. The practice is objectionable and sometimes dangerous. The courts have held that a physician who abbreviates in his prescriptions, is guilty of contributory negligence, in case the dispenser makes an error. If, for example, a physician prescribes hydrarg chlorid intending hydrargyri chloridum mite and the druggist dispenses hydrargyri chloridum corrosivum, both physician and druggist could be convicted of manslaughter if the error should cause the death of the patient.

